# Relapsing polychondritis in childhood: A case report

**DOI:** 10.1097/MD.0000000000040106

**Published:** 2024-10-11

**Authors:** Peng Li, Zhipeng Chen, Huaiqing Lv, Liqiang Lin

**Affiliations:** a School of Clinical Medicine, Shandong Second Medical University, Weifang, China; b Department of Otorhinolaryngology, Linyi People’s Hospital, Linyi, China.

**Keywords:** pediatric, relapsing polychondritis, respiratory involvement, tracheostomy

## Abstract

**Rationale:**

Relapsing polychondritis (RP) is a rare immune-mediated disease that leads to progressive cartilage destruction, notably affecting the ears, nose, and airways. Timely diagnosis is essential to prevent irreversible airway damage and life-threatening complications. This study presents a case of a 12-year-old child diagnosed with RP, emphasizing the critical need for timely identification of RP in children.

**Patient concerns:**

A 12-year-old girl was admitted with worsening hoarseness and cough, leading to respiratory distress and severe bradycardia, requiring transfer to the pediatric intensive care unit. After successful resuscitation and tracheal intubation, imaging showed significant subglottic swelling. She received antibiotics, corticosteroids, and nebulization. Despite initial stabilization, she required a tracheostomy 2 months post-discharge due to recurrent dyspnea. Six months later, she developed joint pain and a saddle nose deformity.

**Diagnoses:**

The patient was clinically diagnosed with RP in accordance with the diagnostic standards set forth by Micheet et al.

**Interventions:**

During the initial phase of treatment, the patient was administered antibiotics, corticosteroids, and nebulization therapy. Due to severe respiratory distress, an emergency tracheostomy was performed by ear, nose, and throat surgeons. After a definitive diagnosis, the patient was treated with tocilizumab, methotrexate, and corticosteroid therapy. Additionally, supportive measures including calcium supplementation, gastric protection, and immune support were provided.

**Outcomes:**

Three years post-diagnosis, the patient’s condition is stable.

**Lessons:**

Due to RP low prevalence, diagnosis in children is frequently delayed or overlooked. Notably, involvement of the major airways is more common and severe in pediatric patients compared to adults. It is crucial for Otolaryngologists to have a comprehensive understanding of this condition to effectively diagnose and manage complications associated with RP.

## 1. Introduction

Relapsing polychondritis (RP) is a rare autoimmune disorder first described by Jaksch-Wartenhorst in 1923 as “polychondropathia.”^[1,2]^ The primary pathogenic mechanism of RP is thought to involve an autoimmune reaction to cartilage antigens.^[3]^ This systemic inflammatory disease is characterized by recurrent and progressive inflammation and degeneration of cartilage, primarily affecting the ears, eyes, nose, trachea, larynx, and joints, these changes often lead to significant pain and deformities.^[1]^ The incidence of RP peaks between the ages of 40 and 60.^[4]^ In the pediatric population, RP is exceedingly rare, with fewer than 10% of cases occurring in children and adolescents.^[5]^ Due to its low prevalence, diagnosis in children is frequently delayed or overlooked.^[5]^ Notably, involvement of the major airways is more common and severe in pediatric patients compared to adults.^[1]^ Approximately one-third of RP patients also have another autoimmune disorder.^[2]^

Treating RP is complicated by its recurring episodic nature. Several immunosuppressive medications, including steroids and steroid-sparing disease-modifying antirheumatic drugs such as methotrexate and azathioprine, have been used with varying success.^[1,2]^ This report details a rare case of pediatric-onset RP, presenting with diffuse tracheobronchial narrowing.

## 2. Case report

**Figure 1.**
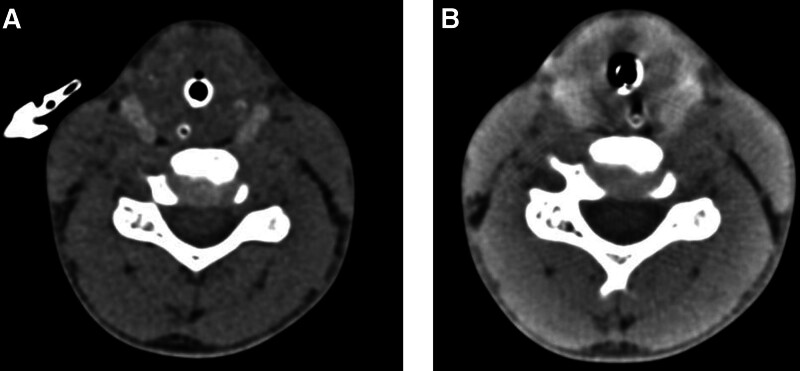
(A) A CT scan of the neck revealed significant bilateral soft tissue swelling in the subglottic region. (B) Follow-up CT imaging of the neck indicated a notable decrease in subglottic soft tissue swelling compared to the previous scan. CT = computed tomography.

A 12-year-old girl was admitted to the pediatric department with a 2-month history of hoarseness and cough that progressed to difficulty breathing. Her symptoms rapidly escalated, leading to her transfer to the pediatric intensive care unit. There, she experienced severe bradycardia, with her heart rate dropping to 0 beats per minute. Immediate cardiopulmonary resuscitation and tracheal intubation were performed, successfully reviving her. A computed tomography scan of the neck revealed significant bilateral soft tissue swelling in the subglottic region (Fig. 1A). The patient received treatment with antibiotics, corticosteroids, and nebulization. Laryngoscopy showed mild bilateral vocal cord edema with normal mobility and subglottic mucosal swelling. Follow-up computed tomography imaging of the neck indicated a notable decrease in subglottic soft tissue swelling compared to the previous scan (Fig. 1B). The patient’s vital signs stabilized, and laboratory findings returned to normal: hemoglobin 125.0 g/L, white blood cell count 7.46 × 10^9^/L, platelet count 371 × 10^9^/L, C-reactive protein 0.44 mg/L, and erythrocyte sedimentation rate 13 mm/h. Other clinical parameters, including coagulation profile, glucose, and renal and liver function tests, were within normal ranges. She was subsequently discharged in stable condition. Despite initial improvements, the patient experienced recurrent dyspnea and underwent a tracheostomy performed by ear, nose, and throat surgeons 2 months after discharge. Six months later, she developed painful edema in her left knee and a saddle nose deformity. Rheumatologic tests conducted at an external hospital were negative, with normal C3 and C4 levels. Based on the McAdam criteria,^[6]^ she was diagnosed with relapsing polychondritis.

Over the next 3 years, she has been under our care, receiving tocilizumab, methotrexate, and corticosteroids, along with calcium supplements, gastric protection, and immune support. Currently, the patient remains in stable condition.

The study was conducted with the written informed consent of the guardians of the underage participant.

Data sharing is not applicable to this article as no datasets were generated or analyzed during the current study.

## 3. Discussion

RP is a rare, progressive autoimmune disorder primarily characterized by recurring episodes of cartilage inflammation and degeneration in the joints, larynx, trachea, ears, and nose.^[2]^ In addition to these primary features, RP can also present with various other clinical symptoms, including vasculitis, myocarditis, ocular inflammation, audiovestibular dysfunction, and nonarthritis.^[2]^ Notably, approximately one-third of RP patients also have another autoimmune disorder.^[2]^ The incidence of RP is quite low, estimated at about 3.5 cases per 1,000,000 individuals.^[7]^ The disease predominantly affects those aged 40 to 60 years and is more frequently observed in middle-aged Caucasians.^[3,7]^ Pediatric cases of RP are exceedingly rare, accounting for <5% of reported cases.^[8]^

Importantly, women are 3 times more likely than men to develop severe airway manifestations of RP.^[7]^ Although the precise pathophysiological mechanisms of RP remain elusive, it is generally thought to be associated with an autoimmune reaction to type II collagen.^[2]^ Type II collagen antibodies are present in approximately one-third of patients with RP.^[7]^

The diagnosis of RP follows criteria established by Micheet et al, which stipulate that inflammation must affect at least 2 of 3 sites: auricular, nasal, or laryngotracheal cartilage, or any one of these sites with at least 2 other manifestations such as ocular inflammation (conjunctivitis, keratitis, episcleritis, or uveitis), hearing loss, vestibular dysfunction, and nonerosive arthritis.^[9]^ Currently, specific laboratory tests for definitive RP diagnosis are unavailable.^[9]^ Studies show that RP diagnosis in pediatric patients is often delayed by up to 5 years, whereas in adults, diagnostic delay is typically <2 years.^[8]^

RP typically affects cartilage tissues in the ears (85% of cases) and nose (62% of cases).^[9]^ Musculoskeletal manifestations, observed in 50% to 75% of patients, usually affect parasternal joints (sternoclavicular, costochondral, and manubriosternal articulations) and peripheral joints, with manifestations generally nonerosive and exhibit asymmetrical patterns.^[9]^ Airway involvement is reported in approximately 50% of RP cases, with associated symptoms such as dyspnea, cough, wheezing, stridor, hoarseness, and aphonia.^[7]^ Compared to adults, young patients appear to experience laryngotracheobronchial tree chondritis more frequently and severely, which may increase the necessity for tracheostomy.^[9]^ Airway involvement is the primary cause of morbidity and mortality in RP.^[7]^ The prognosis of RP varies, depending on the specific organs involved and the patient’s response to treatment, on average, the mortality rate for RP patients is more than twice that of the general population.^[2]^

Currently, there is no standardized treatment protocol for RP.^[7]^ In the absence of randomized therapeutic trials, treatment remains largely empirical. Corticosteroids are typically first-line therapy, for patients with persistent or refractory disease, immunosuppressive agents such as cyclophosphamide, azathioprine, cyclosporine, methotrexate, and mycophenolate mofetil are commonly used as steroid-sparing agents.^[5]^ Additional supportive medications are crucial to mitigate the adverse effects associated with long-term steroid therapy.^[5]^

Several studies, including the case we are reporting, have demonstrated that tocilizumab significantly alleviates clinical symptoms of RP, showing substantial efficacy.^[10–13]^ Infliximab has also been reported to yield a favorable therapeutic response in RP.^[5]^ Complete remission has been reported in patients with severe airway RP following autologous stem cell transplantation or administration of oral bovine type II collagen.^[7]^ However, given RP’s relapsing–remitting nature, the reported successes of these novel treatments may be coincidental.^[7]^ Thus, there is an urgent need for clinical trials and longer follow-ups to rigorously assess the efficacy of these potential therapies.^[7]^

This study is confined by several limitations. The most notable is the solitary case study, which impedes the extrapolation of our results to a broader population. Our diagnostic approach was clinical, lacking the support of specific laboratory confirmation. The durability of the patient’s favorable response to treatment with tocilizumab, methotrexate, and corticosteroids is unconfirmed and requires future longitudinal studies for validation. Nonetheless, our findings contribute meaningfully to the clinical understanding and management of pediatric relapsing polychondritis.

## 4. Conclusion

In conclusion, we present a rare case of pediatric RP. It is crucial for Otolaryngologists to have a comprehensive understanding of this condition to effectively diagnose and manage complications associated with RP.

## Acknowledgments

Thanks to Linyi People’s Hospital for their support to write this case report.

## Author contributions

**Conceptualization:** Peng Li, Zhipeng Chen, Huaiqing Lv, Liqiang Lin.

**Investigation:** Peng Li, Zhipeng Chen, Liqiang Lin.

**Methodology:** Liqiang Lin.

**Project administration:** Peng Li.

**Resources:** Peng Li.

**Supervision:** Peng Li, Zhipeng Chen, Liqiang Lin.

**Validation:** Peng Li, Liqiang Lin.

**Writing – original draft:** Peng Li, Zhipeng Chen, Huaiqing Lv.

**Writing – review & editing:** Peng Li, Liqiang Lin.

## References

[1] Alqanatish JT Alshanwani JR. Relapsing polychondritis in children: a review. Mod Rheumatol. 2020;30:788–98. 31858851 10.1080/14397595.2019.1707995

[2] Figaro NJ Figaro KA Juman JS Arozarena R Davis King K Juman S. Pediatric-onset relapsing polychondritis with otolaryngeal manifestations. Cureus. 2023;15:e40085. 37425495 10.7759/cureus.40085 PMC10327613

[3] Fonseca AR de Oliveira SK Rodrigues MC Aymoré IL Domingues RC Sztajnbok FR. Relapsing polychondritis in childhood: three case reports, comparison with adulthood disease and literature review. Rheumatol Int. 2013;33:1873–8. 22210275 10.1007/s00296-011-2336-6

[4] Knipp S Bier H Horneff G. Relapsing polychondritis in childhood—case report and short review. Rheumatol Int. 2000;19:231–4. 11063294 10.1007/s002960000055

[5] Alqanatish JT Alfarhan BA Qubaiban SM. Limited auricular relapsing polychondritis in a child treated successfully with infliximab. BMJ Case Rep. 2019;12:e227043. 10.1136/bcr-2018-227043 PMC6536242 31126928

[6] Winter G Löffelmann T Chaya S. Relapsing polychondritis with tracheobronchial involvement: a detailed description of two pediatric cases and review of the literature. Klin Padiatr. 2024;236:97–105. 38224687 10.1055/a-2230-1521 PMC10883755

[7] Gorard C Kadri S. Critical airway involvement in relapsing polychondritis. BMJ Case Rep. 2014;2014:bcr2014205036. 10.1136/bcr-2014-205036 PMC4166125 25213785

[8] Belot A Duquesne A Job-Deslandre C. Pediatric-onset relapsing polychondritis: case series and systematic review. J Pediatr. 2010;156:484–9. 19880136 10.1016/j.jpeds.2009.09.045

[9] Buscatti IM Giacomin MF Silva MF Campos LMA Sallum AME Silva CA. Laryngotracheal stenosis requiring emergency tracheostomy as the first manifestation of childhood-relapsing polychondritis. Acta Reumatol Port. 2013;38:208–11. 24149019

[10] Kirino Y Takase-Minegishi K Tsuchida N. Tocilizumab in VEXAS relapsing polychondritis: a single-center pilot study in Japan. Ann Rheum Dis. 2021;80:1501–2. 34260381 10.1136/annrheumdis-2021-220876

[11] Farhat R Clavel G Villeneuve D. Sustained remission with tocilizumab in refractory relapsing polychondritis with ocular involvement: a case series. Ocul Immunol Inflamm. 2021;29:9–13. 32643976 10.1080/09273948.2020.1763405

[12] Wendling D Godfrin-Valnet M Prati C. Treatment of relapsing polychondritis with tocilizumab. J Rheumatol. 2013;40:1232. 10.3899/jrheum.130371 23818726

[13] Liao HT. Efficacy of tocilizumab for refractory relapsing polychondritis with tracheal stenosis and respiratory distress. Rheumatology (Oxford). 2022;61:1293–4. 33989404 10.1093/rheumatology/keab442

